# PCr/ATP ratios and mitochondrial function in the heart. A comparative study in humans

**DOI:** 10.1038/s41598-023-35041-7

**Published:** 2023-05-23

**Authors:** Vera H. W. de Wit-Verheggen, Vera B. Schrauwen-Hinderling, Kim Brouwers, Johanna A. Jörgensen, Gert Schaart, Anne Gemmink, Emmani B. M. Nascimento, Matthijs K. C. Hesselink, Joachim E. Wildberger, Patrique Segers, David Montaigne, Bart Staels, Patrick Schrauwen, Lucas Lindeboom, Joris Hoeks, Tineke van de Weijer

**Affiliations:** 1 Department of Nutrition and Movement Sciences, School of Nutrition and Translational Research in Metabolism, Maastricht University Medical Center, Universiteitssingel 50, P.O. Box 616, 6200 MD Maastricht, The Netherlands; 2 Department of Radiology and Nuclear Medicine, Maastricht University Medical Center, 6200 MD Maastricht, The Netherlands; 3 Department of Cardiothoracic Surgery, Maastricht University Medical Center, 6200 MD Maastricht, The Netherlands; 4 University Lille, Inserm, CHU Lille, Institut Pasteur de Lille, U1011-EGID, F-59000 Lille, France

**Keywords:** Magnetic resonance imaging, Metabolic diseases, Mitochondria

## Abstract

Cardiac energy status, measured as phosphocreatine (PCr)/adenosine triphosphate (ATP) ratio with 31P-Magnetic Resonance Spectroscopy (31P-MRS) in vivo, is a prognostic factor in heart failure and is lowered in cardiometabolic disease. It has been suggested that, as oxidative phosphorylation is the major contributor to ATP synthesis, PCr/ATP ratio might be a reflection of cardiac mitochondrial function. The objective of the study was to investigate whether PCr/ATP ratios can be used as in vivo marker for cardiac mitochondrial function. We enrolled thirty-eight patients scheduled for open-heart surgery in this study. Cardiac 31P-MRS was performed before surgery. Tissue from the right atrial appendage was obtained during surgery for high-resolution respirometry for the assessment of mitochondrial function. There was no correlation between the PCr/ATP ratio and ADP-stimulated respiration rates (octanoylcarnitine R^2^ < 0.005, p = 0.74; pyruvate R^2^ < 0.025, p = 0.41) nor with maximally uncoupled respiration (octanoylcarnitine R^2^ = 0.005, p = 0.71; pyruvate R^2^ = 0.040, p = 0.26). PCr/ATP ratio did correlate with indexed LV end systolic mass. As no direct correlation between cardiac energy status (PCr/ATP) and mitochondrial function in the heart was found, the study suggests that mitochondrial function might not the only determinant of cardiac energy status. Interpretation should be done in the right context in cardiac metabolic studies.

## Introduction

Magnetic resonance spectroscopy (MRS) is a non-invasive imaging technique that has the potential to quantify metabolites in vivo. Using Phosphorus MRS (^31^P-MRS), a spectrum containing signals of high energy, phosphorus containing metabolites in the cardiac muscle can be acquired. The most abundant phosphorous containing metabolites in the human heart are adenosine triphosphate (ATP) and phosphocreatine (PCr). The ratio between PCr and ATP (the PCr/ATP ratio) has been applied in human studies. Here it was shown that participants with T2DM^1–4^ and heart failure^5^ have reduced cardiac PCr/ATP ratios compared to healthy controls. Also, in heart failure, the PCr/ATP ratio has been shown to be of prognostic value^6^. But what does a lowerd PCr/ATP ratio represent, and how should it be interpreted?

In the cardiomyocyte, PCr is buffering ATP concentration when ATP demand is increased, the PCr/ATP ratio reflects the myocardial energy status. As ATP production in the heart is almost entirely driven by mitochondrial oxidative metabolism, a low PCr/ATP ratio may be a marker of compromised mitochondrial function in cardiac tissue.

In parallel to the human studies showing decreased PCr/ATP ratio’s in type 2 diabetes^1–4^ and heart failure^5^, animal studies have reported a lower cardiac mitochondrial respiration in permeabilized cardiac muscle fibers in T2DM and heart failure^6–8^. These data may suggest that indeed an impaired cardiac energy metabolism, indicated by a reduction of the myocardial PCr/ATP ratio, may be linked to ex vivo measured cardiac mitochondrial dysfunction. However, the direct relation between PCr/ATP ratio and cardiac mitochondrial function is not yet established. Several factors, such as creatine supply, pH, oxygen supply, CK activity and instant myocardial work may independently influence either PCr concentrations or mitochondrial function in the cardiomyocyte. Hence, it remains unclear to what extent PCr/ATP can be used as a marker of mitochondrial function^9,10^.

Therefore, the aim of the present study was to investigate whether in vivo PCr/ATP ratios measured in the left ventricle (LV) with ^31^P-MRS, as a non-invasive readout, correlate with ex vivo mitochondrial respiratory capacity, measured in permeabilized myocardial fibers.

## Methods

### Participants

Recruitment and data collection took place between March 2017 and March 2019 in The Maastricht University Medical Center, The Netherlands. Thirty-eight participants (male and female) between 40 and 75 years, who were scheduled to undergo an open-heart surgery with extracorporeal circulation enrolled in the study. The included surgical procedures were coronary artery bypass grafting, and/or aortic (valve) surgery, and/or mitral valve surgery.

The current sample size calculation was based on clinically relevant associations PCr/ATP (measured by 31P-MRS) and maximal mitochondrial capacity (measured by high-resolution respirometry). Therefore, we performed a sample size calculation for linear regression analysis (with a single predicting variable). To be of biological/clinical significant a correlation factor of at least 0.4–0.5 would be expected. A correlation coefficient is directly related to a regression coefficient, and thus the sample size calculation was determined based on an expected correlation coefficient of 0.45. To reach a power (1 − β) of 80% and a significance level (α) of 5% with two-sided testing, a minimal sample size of patients was determined to be 33. To be on the safe side, with an expected drop-out during the study of 20%, we included 38 patients. As we know that mitochondrial function and the PCr/ATP ratios are lowered in obese and T2DM subjects, we included both lean, obese and T2DM to ensure spread in the data to facilitate correlation analysis. Inclusion criteria were sinus node rhythm, stable dietary habits (no weight change of more than 3 kg in the last 6 months), and stable physical activity levels for at least 6 months. Exclusion criteria were active diseases (besides cardiovascular disease or T2DM), and contra-indications for magnetic resonance imaging (MRI). The enrolled study population encompassed participants with a wide BMI range from 19.4 to 37.2 kg/m^2^. T2DM patients had to be diagnosed for more than 2 years, have a BMI above 27 kg/m^2^, be (moderately) well-controlled (defined by a HbA1c < 8%), had to be non-insulin dependent, and had to be on a dietary treatment or on sulphonylurea (SU)-derivate or metformin therapy for at least six months with a constant dose for at least 2 months.

### Ethical approval

The study was conducted in accordance with the principles of the declaration of Helsinki and approved by the Ethics Committee of the Maastricht University Medical Center. All participants provided written informed consent. The study was registered at https://clinicaltrials.gov (NCT03049228).

### Study design

In this observational study, prior to the scheduled cardiothoracic surgery, participants visited the Maastricht University Medical Center for several measurements: resting energy metabolism, fasted blood sampling, body composition, MRI, and MRS. Participants received a standardized meal the evening before the tests and stayed fasted from 8 PM onwards. Participants were instructed to refrain from strenuous exercise and alcohol in the last three days prior to the tests. During the scheduled surgical procedure, a tissue specimen of the right atrial appendage tissue was taken (average time delay between the test day and surgery was 14.1 ± 12.1 days).

### Resting energy metabolism

After an overnight fast, oxygen consumption and carbon dioxide production was measured for 30 min with an automated respiratory gas analyzer, using a ventilated hood system (Omnical, IDEE Maastricht, The Netherlands). Calculations of substrate oxidation and energy expenditure, were computed with the assumption of a negligible protein oxidation^11^.

### Body composition

Body mass and body volume were assessed using air-displacement plethysmography using the Bod Pod device (Cosmed, Italy, Rome) according to the manufacturer’s instructions^12^. Thoracic gas volume was predicted on equations included in the Bod Pod software. From these data, body composition (including body fat percentage) was calculated as described by Siri et al.^13^.

### ^31^P-MRS acquisition

After an overnight fast, the in vivo cardiac energy status was determined. All measurements were performed on a 3.0 T whole body MRI scanner (Achieva 3 T-X, Philips Healthcare, Best, The Netherlands). The coil that was used was a dual tuned, custom-made cardiac 1H/31P coil with a 31P-transmit loop of 28 cm diameter and a somewhat smaller receive loop of 18 cm. The 1H part was receive-only with a diameter 23.5 cm.

**Figure 1:**
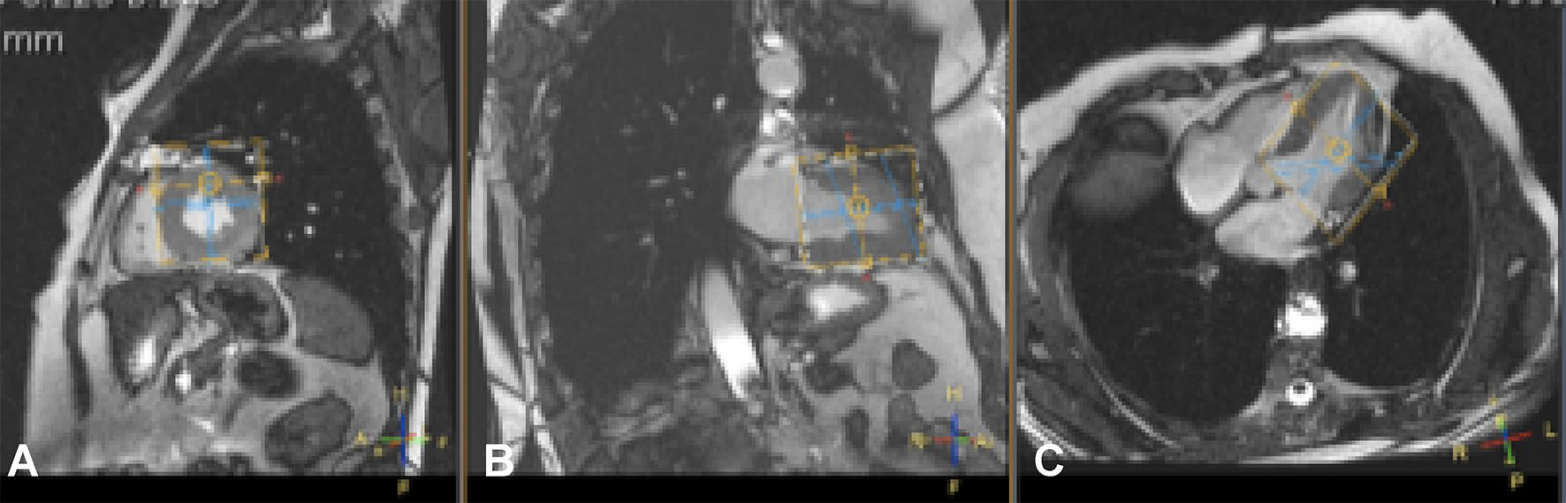
Volume of interest to acquire a cardiac ^31^P-MRS spectrum. The volume of interest (yellow) is carefully placed in maximum systole to ensure the myocardium of the LV is taken into account, without muscle mass of the pectoralis and diaphragm, and minimizing the volume that is placed in the lungs. (**A**) sagittal view, (**B**) coronal view, (**C**) transversal view.

Here, the myocardial PCr to ATP ratio was quantified by ^31^P-MRS, using a 3D ISIS sequence. Participants were positioned prone and head first in the MRI scanner. A double tuned ^1^H and ^31^P surface cardiac coil (Rapid Biomedical, Rimpar, Germany) was placed under the participant’s chest. After making scout-images of the heart, the volume of interest was carefully placed over the total volume of the LV of the heart (Fig. 1). Spectra were acquired in expiration during the end-systolic phase (using ECG-triggering, NSA = 96, number of points = 2048, bandwidth = 3000 Hz). We used a 90 degree block pulse for excitation with a BW of 4111 Hz (calibrated to a distance of 8 cm, typical for the heart), centred to the middle between PCr and γ-ATP, NSA = 128 (which equals to 16 full 3D-ISIS acquisitions), number of points = 2048. This was according to the protocol validated by Lamb et al.^14^. The Measurement was centred over the Left ventricle with spacing for the thoracic wall. no frequency offset, NSA = 128 (which equals to 16 full 3D-ISIS acquisitions). Measurements were performed twice, with a repetition time of 5 heartbeats and 8 heartbeats and volunteers were asked to breathe in the rhythm of the measurements. Respiration was monitored during the whole measurements with a respiratory sensor and continuous breathing instructions were used to make sure that volunteers were in the exhaled position during spectral acquisition. Breathing instructions were given when necessary.

### ^31^P-MRS spectral post-processing

**Figure 2:**
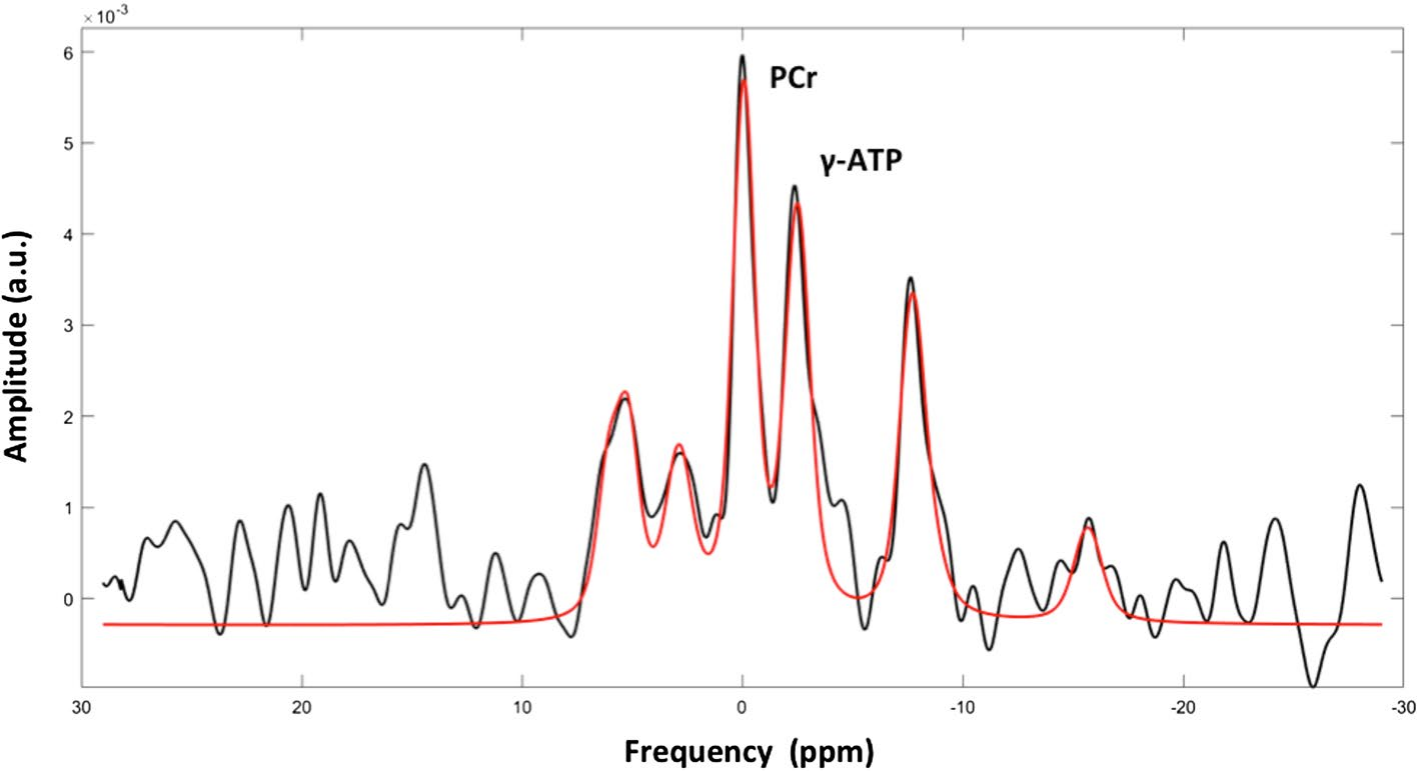
Cardiac ^31^P-MRS spectrum. Participant with PCr/ATP ratio = 1.239. In black the original cardiac 31P spectrum with first order phase adjustment and in red the fit of the high energy phosphorus metabolite peaks Pi, PDE, 2,3DPG, PCr, γ-ATP, and α-ATP, at their typical resonance frequency. The resonances of PCr and γ-ATP were considered for calculation of PCr/ATP.

PCr and ATP resonances were quantified using a custom written MATLAB (MATLAB 2014b, The MathWorks, Inc., Natick, Massachusetts, United States; https://.mathworks.com/) script. In the fitting algorithm, the time domain signal was simulated based on prior knowledge from literature. The amplitudes and relative frequency shifts of the individual peaks were updated iteratively according to the routine used in Roumans et al.^15^. In each step, the difference between the simulated spectrum and the acquired spectrum was minimized (first for a rough fit in the time domain and then in the frequency domain, where the fit focused specifically on the region of PCr and ATP). Next to the amplitude and relative frequency shift, also the Gaussian line broadening, the Lorentzian line broadening, the zero order phase, an overall frequency shift and the baseline offset were automatically updated, affecting all the peaks in an identical fashion. Values were corrected for T1 saturation (PCr 5.8 s and gamma-ATP 3.1 s according to El-Sharkawy et al.^16^) and corrected for the ATP contribution from blood^17^. In some samples the 2.3-DPG signal was very small or even missing, leading to a generally low blood correction in the current study. Values are expressed as ratio of PCr over gamma-ATP (PCr/ATP). To quantify PCr and ATP, a spectrum was simulated (based on the known delay excitation and acquisition). The spectra were fitted in the time domain, however, no first order phase was applied. Zero order phase was automatically determined and the real spectra (not magnitude) were used for fitting. A simulated spectrum with resonances at known frequencies was used and the amplitudes of the various resonances was modulated iteratively in order to minimize the residual. Damping of the signals (corresponding to the linewidth in the frequency domain) were varied in the same fashion for all resonances until the residual was minimal. The delay between excitation and acquisition was the same for all measurements and this delay was taken into account in the simulated spectrum used for fitting. For displaying reasons only, a first order phase was applied to compensate for the delay between excitation and acquisition. This is shown in Fig. 2. Only 35 participants could be included in the analysis of cardiac energy status.

The quality of the spectra and the fit was independently scored by two experienced spectroscopists in a blinded fashion. The spectra were visually rated by the senior spectroscopist in a blinded fashion. Spectra were categorized into three categories (good, sufficient and insufficient quality) and the spectra of good and sufficient quality were included. Reasons for exclusion was inappropriate fit (high residual), low SNR (by visual inspection) or significant artefacts. In order to determine the reproducibility of the measurements, 8 participants underwent a repeated measurement resulting in a coefficient of variation of 11.8 ± 6.9% for a repetition time of 5 heartbeats (n = 3) and 4.4 ± 2.5% for a repetition time of 8 heartbeats (n = 5).

In 20 participants, both spectra (with a repetition time of 5 heartbeats and of 8 heartbeats) were of good quality and allowed comparison between the two acquisition schemes. A 2-tailed paired sample t-test showed that the PCr/ATP ratios at the different repetition times were similar (0.97 ± 0.29 and 0.93 ± 0.28, p = 0.425). Therefore, and as the reproducibility of both repetition times was good, the average of the spectra from different repetition times was reported when both were available (n = 20) and either the long or the short repetition time is reported if one of the two was lost due to insufficient quality.

### Cardiac function with MRI

During the ^31^P-MRS acquisition a cardiac CINE-MRI examination was performed and analysed with dedicated software (CAAS, PieMedical Imaging version 1.0 2018, Maastricht, The Netherlands) to determine LV end diastolic and LV end systolic volumes, LV end systolic mass, and LV ejection fraction as previously described^18^. Parameters were corrected for body surface area^19^ according to Haycock^20^. Due to technical failures, cardiac function is only obtained in 32 subjects.

### Cardiac biopsy

A cardiac biopsy from the right atrial appendage tissue was collected under general anesthesia (Sufentanil, Midazolam, Rocuronium, Propofol) and with extracorporeal circulation. Due to complications during surgery, tissue sampling was not possible in 1 participant. Immediate tissue handling was performed in the operation room and cardiac tissue was directly separated from blood and visible fat. Part of the biopsy was immediately placed in ice-cold preservation medium (BIOPS, OROBOROS Instruments, Innsbruck, Austria) and used for the preparation of permeabilized cardiac fibers and subsequent measurement of ex vivo mitochondrial oxidative capacity. Remaining cardiac tissue was used for the measurement of structural sub-units of the 5 complexes in the electron transport chain (OxPhos) by Western blotting (AB110411, Abcam, Cambridge, UK) as described in Ref.^21^.

### Mitochondrial respiration rates (high resolution respirometry)

Cardiac tissue fibers were permeabilized with saponin according to the protocol of Lemieux et al.^7^. After completion of the permeabilization protocol, cardiac muscle fibers were transferred into ice-cold mitochondrial respiration buffer (MiR05, OROBOROS Instruments, Innsbruck, Austria) for assessment of oxygen consumption rate, using high-resolution respirometry (OROBOROS Oxygraph-2 k, Innsbruck, Austria).

Briefly, two multiple substrate-uncoupler protocols, stimulating different complexes of the electron transport chain of the mitochondria were performed at 37ºC and in quadruplicate. Oxygen limitation of fibers was avoided by maintaining oxygen levels between 200 and 400 μmol/l O_2_. In every protocol applied, first, 4.0 mmol/l malate was added, followed by 1.0 mmol/l octanoylcarnitine (trace 1) or 5.0 mmol/l pyruvate (trace 2) ), for the assessment of basal resting respiration rates. In addition, an excess of 2.0 mmol/l ADP was added to evaluate state 3 respiration (ADP stimulated respiration). Then 10.0 mmol/l glutamate was added as an additional substrate for complex I after which 10.0 mmol/l succinate was added to obtain state 3 respiration fueled by both complex I and II. Finally, the chemical uncoupler fluoro-carbonyl cyanide p-trifluoromethoxyphenylhydrazone (FCCP) was titrated to evaluate maximal respiratory capacity (state U). The integrity of the outer mitochondrial membrane was assessed by the addition of 10.0 μmol/l cytochrome C upon maximal coupled respiration. Datlab software (OROBOROS Instruments, Innsbruck, Austria) was used for data acquisition and analysis. Oxygen consumption measurements took place within 2.7 ± 0.7 h after surgery for the first protocol, and within 4.5 ± 1.3 h for the second protocol. This timeframe has been shown not to affect OXPHOS capacity^7^. The amount of sampled tissue was not always sufficient to perform both protocols in each participant. Therefore, protocol 1 was performed 35 participants, and protocol 2 was performed in 36 participants.

### Blood sampling and analyses

Glucose, FFA, triglycerides, and cholesterol were analyzed using a Pentra 400 (Horiba, Montpellier, France). All samples from one participant were analyzed within one run. HbA1c and HDL-cholesterol were determined by the hospital laboratory, and LDL-cholesterol was calculated according the Friedewald formula.

### Statistics

Data are presented as mean ± SEM (standard error of the mean) unless indicated otherwise. Linear regression analyses were performed to identify correlations between variables. Group comparisons were assessed by one-way ANOVA. In case of significance, Bonferroni adjusted post-hoc analyses were applied to determine group specific differences. After confirming a normal distribution of the data using a Shapiro–Wilk test, Pearson correlation coefficient was determined for binary correlation analysis*.* Statistical significance was set at a p-value < 0.05. Statistical analyses were performed with the use of IBM Statistical Package for Social Sciences for MAC, version 23 (SPSS, Inc.).

## Results

### Participant characteristics

Thirty-eight men and women (age 64.8 ± 6.7 years; 24% female) participated in the study. Participant characteristics are reported in Table 1.

**Table 1:** Participant characteristics (n = 38).

	Participants
Sex (% female)	24 ± 4
Age (years)	64.8 ± 6.7
Smoking status (% yes)	29 ± 5
Type of cardiac surgery* (%)	
Coronary artery bypass grafting	74
Aortic valve replacement	24
Mitral valve replacement	11
Aortic (root) surgery	21
BMI (kg/m^2^)	28.4 ± 3.9
Fat mass (kg)	30.1 ± 10.2
Fat free mass (kg)	53.8 ± 10.0
Fat percentage (%)	35.5 ± 8.7
Fat free percentage (%)	64.5 ± 8.7
HbA1c (%)	6.1 ± 1.0
Fasting glucose (mmol/L)	7.0 ± 2.0
Total cholesterol (mmol/L)	3.8 ± 1.5
HDL cholesterol (mmol/L)	1.31 ± 0.39
LDL cholesterol (mmol/L)	2.26 ± 1.03
Triglycerides (mmol/L)	1.58 ± 1.06
Free fatty acids (μmol/L)	688 ± 250
Use of lipid lowering drugs (% yes)	82 ± 4
Respiration quotient	0.79 ± 0.05
Carbohydrate oxidation (g/min)	0.10 ± 0.06
Fat oxidation (g/min)	0.09 ± 0.03
Energy expenditure (kJ/min)	5.04 ± 0.77

Data are presented as means ± standard deviation, or as proportions (%).

*In some cases the surgical interventions were combined; for instance aortic root surgery was sometimes combined with aortic valve replacement, and in some cases a coronary artery bypass grafting may have been combined with valve replacement surgery.

### In vivo cardiac PCr/ATP ratio and the correlation with ex vivo cardiac mitochondrial function

**Figure 3:**
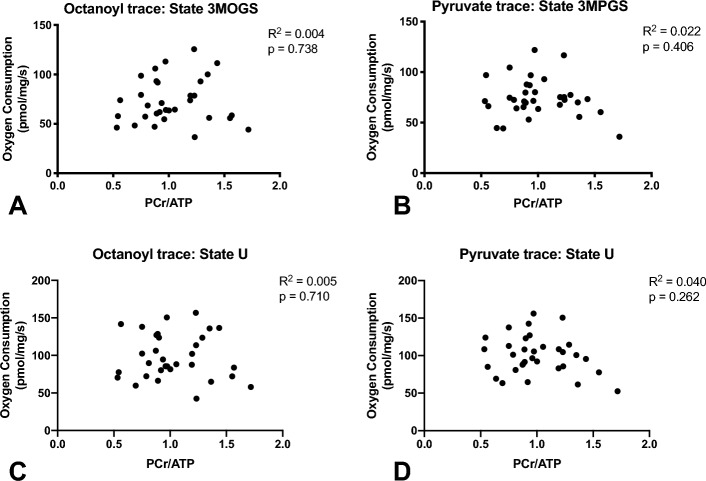
No correlation between PCr/ATP and mitochondrial respirometry. PCr/ATP did not correlate with ADP-stimulated respiration, neither upon (**A**) malate, octanoylcarnitine, glutamate, succinate nor upon (**B**) malate, pyruvate, glutamate, succinate. PCr/ATP did also not correlate with maximally uncoupled respiration, neither upon (**C**) malate, octanoylcarnitine, glutamate, succinate nor upon (**D**) malate, pyruvate, glutamate, succinate.

**Figure 4:**
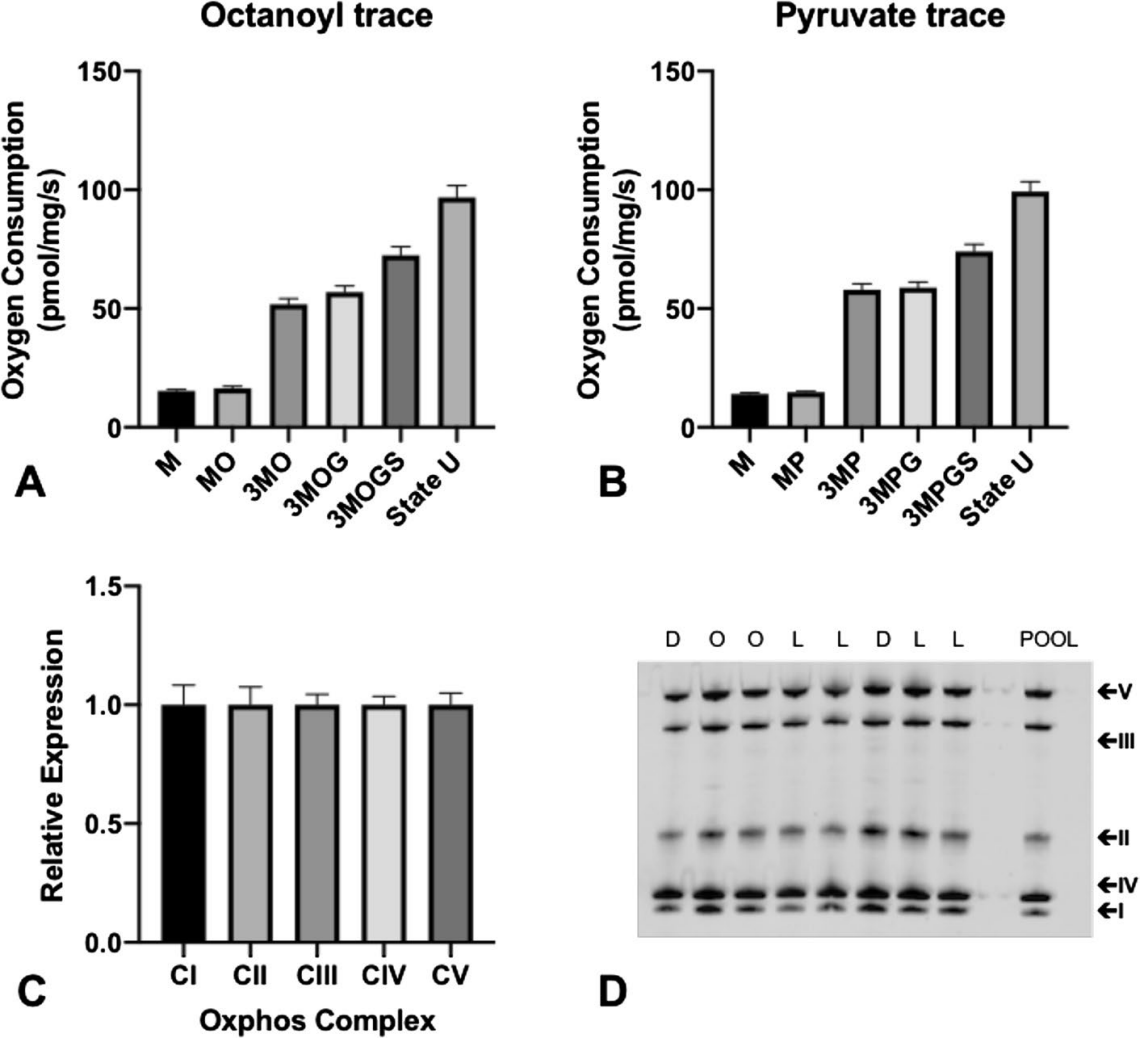
Mitochondrial oxidative capacity and mitochondrial respiratory chain proteins in permeabilized muscle fibers of the right atrial appendage tissue. Respiration measured after addition of malate (state 2, M), (**A**) octanoyl (state 2, MO) or (**B**) pyruvate (state 2, MP), ADP (state 3, 3MO), glutamate (state 3, 3MOG), succinate (state 3, 3MOGS), and FCCP (maximal uncoupled respiration, state U). Data depicts oxygen consumption per mg wet muscle weight per second and is presented as mean ± SEM (standard error of the mean). (**C**) Relative expression of the protein levels of the OXPHOS complexes. (**D**) Representative western blot from 8 participants depicting the oxidative phosphorylation complexes.

To test whether the in vivo cardiac PCr/ATP ratio reflects cardiac mitochondrial function, PCr/ATP was correlated with all respiration states on octanoylcarnitine (lipid-derived substrate, N = 34) as well as on pyruvate (glucose-derived substrate N = 35) (Fig. 3). Normal distribution of the data was checked using a Shapiro-Wilks test. All data were normally distributed. A Pearson correlation coefficient was used for binary correlation analysis. There were no statistically significant relationships between PCr/ATP ratios and the different respiration rates (for all p > 0.05). Thus, ADP-stimulated (state 3) respiration did not correlate with PCr/ATP ratio upon glutamate, succinate or octanoyl carnitine (R^2^ < 0.005, p = 0.74), nor on pyruvate (R^2^ < 0.025, p = 0.41). Furthermore, no correlations between PCr/ATP ratios and maximally uncoupled respiration were found (octanoyl R^2^ = 0.005, p = 0.71, pyruvate R^2^ = 0.040, p = 0.26). Protein levels of the 5 OXPHOS complexes (I – V) did not correlate with in vivo nor with ex vivo measurements (p ≥ 0.05) (Fig. 4).

As atrial fibrosis is frequently being observed in cardiac disease and might severely lower mitochondrial function, participants were checked for the absence of visually detectable fibrosis using a table-top preparation microscope.

### Cardiac function

Cardiac function parameters as measured with MRI are reported in Table 2. Since male and female participants have different reference values^19^, data are presented for male and female participants separately.

**Table 2:** Left ventricular function parameters.

	Male (n = 23)	Female (n = 9)
End diastolic volume (ml)	190.3 ± 71.7	135.6 ± 29.4
End diastolic volume/BSA (ml/m^2^)	93.0 ± 35.4	75.5 ± 17.5
End systolic volume (ml)	79.5 ± 52.8	37.0 ± 11.9
End systolic volume/BSA (ml/m^2^)	38.7 ± 25.1	20.8 ± 7.2
Ejection fraction (%)	60.5 ± 12.3	72.7 ± 7.5
End systolic mass (g)	206.1 ± 40.0	162.0 ± 44.4
End systolic mass/BSA (g/m^2^)	100.8 ± 20.0	89.4 ± 22.0

*BSA* body surface area.

Data are presented as means ± standard error of the mean, or as proportions (%).

Ventricular mass is measured excluding papillary muscle mass.

**Figure 5:**
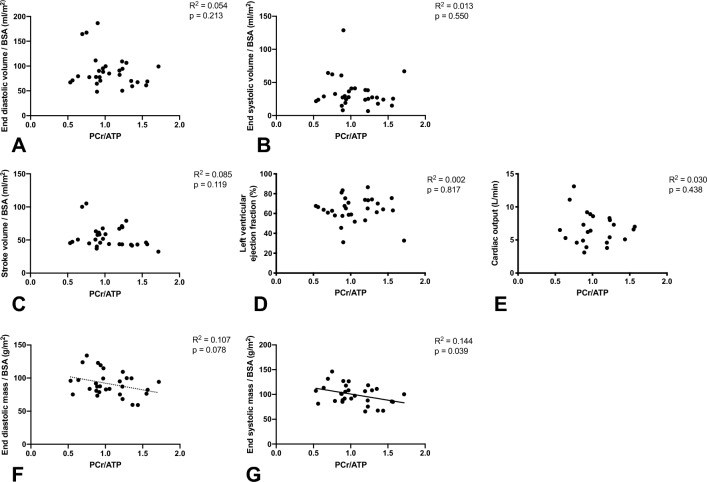
PCr/ATP is correlated with indexed LV end systolic mass. PCr/ATP did not correlate with indexed LV end diastolic (**A**) nor systolic (**B**) volume. There is also no correlation between PCr/ATP and LV Stroke volume (**C**), LV ejection fraction (**D**) nor Cardiac output (**E**). PCr/ATP tended to correlate with End diastolic mass (**F**) and correlated statistically significant with indexed LV end systolic mass (**G**).

Interestingly, indexed LV end systolic mass correlated with PCr/ATP ratio (Fig. 5D, p = 0.04). In contrast, we observed no correlations between state 3 or maximally uncoupled mitochondrial respiration with any of the parameters of cardiac function (Fig. 5).

## Discussion

PCr/ATP ratio has been shown to be diminished in T2DM^1–4^ and heart failure^5^ and to have predictive value for cardiovascular morbidity and mortality^5^, while in parallel mitochondrial function is suggested to be hampered in T2DM and heart failure^6–8^. Therefore, decreased mitochondrial function may underlie the decreased PCr/ATP ratio that is found in these patients. Previous work in animal studies also previously showed a strong relationship between mitochondrial function and PCr/ATP ratios^22,23^. Hence, the PCr/ATP ratio may be a good in vivo marker of mitochondrial function. However, the direct relationship with cardiac mitochondrial function has never been established in humans. Here, we determined PCr/ATP ratio and ex vivo mitochondrial function in a population with a wide range of cardiac metabolic health. However, in contrast to our expectations, we did not find any direct correlations between PCr/ATP ratio in vivo and any of the mitochondrial respiration states measured ex vivo.

Although we know that PCr/ATP ratio’s and mitochondrial function are related, apparently, the PCr/ATP ratio is not solely influenced by mitochondrial function. While it has been previously shown, that a severely diminished mitochondrial function impairs ATP generation, and will lead to a low PCr/ATP ratio by relying more heavily on the buffering by PCr to keep ATP concentrations relatively stable^24,25^, the PCr/ATP ratio may also be influenced by additional factors that do not affect mitochondrial function. For example, a decrease in PCr/ATP ratio may partly be explained by variation in creatine availability. If creatine availability is low, this may limit PCr formation and therefore PCr/ATP ratio, while mitochondrial function may remain unaffected. Furthermore, creatine content decreases in heart failure and its level reflects the severity of heart failure^26,27^. Therefore, the time course of diminishing creatine availability runs in parallel with lowering PCr/ATP ratio and it can be suggested that not ATP but creatine availability is limited in heart failure^27–30^. Variations in creatine availability in the current population may have weakened any potential relationship between PCr/ATP ratio and mitochondrial function. However, as we did not measure cardiac creatine content, this remains speculative.

Interestingly, we did find correlations between the PCr/ATP ratio and cardiac function parameters, whereas the direct relationship between these parameters and ex vivo mitochondrial function were lacking. This suggests that PCr/ATP ratio does remain a relevant parameter for cardiac metabolic health, though not necessarily directly reflecting mitochondrial capacity. Indeed, the relationship between cardiac energy status measured with PCr/ATP ratio and cardiac function parameters has been found previously both in diabetes^1,31^ and heart failure^5,32,33^ and seems consistent throughout studies.

The average value of the PCr/ATP ratio, was low in our study (average PCr/ATP ratio was 1.0 ± 0.3) compared to earlier studies (PCr/ATP ratio 1.7–2.3^1,3,34^ in healthy controls; and 1.5^1,3^ or even 1.9^35^ in T2DM, and around 1.6^5^ in patients with dilated cardiomyopathy). Differences in correction for T1 relaxation and fitting routines may partially explain such differences. Furthermore, differences in pulse bandwidth and profile may affect the detection of 2,3DPG, which is generally used for correction of ATP originating from blood. In addition, the scan technique we used, 3D ISIS, is known to produce lower PCr/ATP ratios compared to perpendicular 1D CSI/1D ISIS^36^, which is probably due to less contamination in the 3D ISIS technique from non-cardiac muscle tissue such as diaphragm or chest wall muscle with high PCr content. However, these methodological issues would not hamper the conclusion regarding the lack of correlation between PCr/ATP ratio and mitochondrial function. In addition, we here study a population with known cardiac disease, which may also partly explain a lower cardiac energy status.

### Study limitations

The main limitation of the current study is that we measured PCr/ATP ratio in the LV of the heart, whereas we measured respiratory capacity in tissue of the right atrial appendage. This was the case because respiration measurements in ventricular tissue specimens would require a LV biopsy, which is a very invasive procedure, while the right atrial appendage can be obtained during open heart surgery without any extra risk for the patient. Indeed Lemieux et al.^7^ previously showed that the absolute respiration rates are lower in the right atrium when compared to the left ventricle, however, they also showed that the mitochondrial quality was similar and that thee was good agreement between respiratory capacity of the LV and the right atrial appendage. There showed that a relatively decrased mitochondrial function will be found in both the left ventricle as well as in the right atrium in cardiac disease. In addition, our measurements of mitochondrial oxidative capacity were within normal range compared to literature^7,37^. Therefore, we are confident that the respiratory rates measured in tissue from the right atrial appendage are characteristic for the whole myocardium and therefore reflect respiratory rates in the LV.

The measured PCr/ATP ratio in this study was low compared to previous literature^1,3,34^, as stated earlier, various explanations exists. Specifically, in some of the spectra the 2.3-DPG signal was low or completely missing, leading to a low correction for blood-borne ATP, possibly contributing to the low PCr/ATP ratio reported in this study.

## Conclusion

In this study, we show that in vivo cardiac energy status of the LV does not directly correlate with ex vivo mitochondrial respiratory capacity in the right atrial appendage tissue. Possibly, the relationship is confounded by other parameters. Nonetheless, the value of cardiac energy status remains indisputable in many cardiac pathologies, as it has been shown to be of prognostic value in heart failure^5^ and a low PCr/ATP indicates difficulties of the heart in providing sufficient energy for contraction. Therefore not surprisingly, PCr/ATP was indeed shown to be related to parameters of cardiac function in this study, proving its value in cardiac metabolic disease.

## Supplementary Information

Supplementary Tables.

Supplementary Figures.

## Data Availability

The authors confirm that the data supporting the findings of this study are available within the article and its supplementary material. Raw data that support the findings of this study are available from the corresponding author, upon reasonable request.

## Abbreviations

^31^P-MRS: ^31^P-magnetic resonance spectroscopy
ATP: Adenosine triphosphate
CABG: Coronary artery bypass grafting
FCCP: Fluoro-carbonyl cyanide p-trifluoromethoxyphenylhydrazone
LV: Left ventricle
MRI: Magnetic resonance imaging
MRS: Magnetic resonance spectroscopy
OXPHOS: Oxidative phosphorylation
PCr: Phosphocreatine
T2DM: Type 2 diabetes mellitus
