# Supplementary material for: PCr/ATP ratios and mitochondrial function in the heart. A comparative study in humans
Source: Sci Rep. 2023 May 23;13:8346. doi: 10.1038/s41598-023-35041-7 (PMC10205750; doi:10.1038/s41598-023-35041-7)
Supplement: Supplementary file 1 — Supplementary Tables. [file 41598_2023_35041_MOESM1_ESM.docx]

***Supplementary***

**Table S1.** Mitochondrial respiration in the right atrial appendage tissue in lean, overweight / obese, and T2DM subjects.

|  | Lean | Overweight / Obese | T2DM | P-value |
| --- | --- | --- | --- | --- |
| **Trace 1** | (n=9) | (n=15) | (n=11) |  |
| M | 15.6 ± 1.3 | 16.1 ± 1.2 | 14.0 ± 0.7 | 0.366 |
| MO | 16.6 ± 1.4 | 17.7 ± 1.9 | 14.6 ± 0.5 | 0.385 |
| 3MO | 56.7 ± 6.0 | 53.1 ± 3.6 | 46.2 ± 1.8 | 0.203 |
| 3MOG | 62.7 ± 7.4 | 57.9 ± 4.0 | 50.7 ± 2.8 | 0.255 |
| 3MOGS | 79.9 ± 10.4 | 73.5 ± 5.1 | 64.7 ± 3.7 | 0.299 |
| State U | 101.4 ± 13.3 | 101.1 ± 7.6 | 87.2 ± 5.1 | 0.432 |
| **Trace 2** | (n=10) | (n=15) | (n=11) |  |
| M | 14.4 ± 0.9 | 13.9 ± 0.7 | 14.2 ± 0.5 | 0.896 |
| MP | 15.0 ± 1.1 | 14.6 ± 0.7 | 14.8 ± 0.7 | 0.940 |
| 3MP | 58.0 ± 5.2 | 55.5 ± 4.0 | 61.2 ± 4.2 | 0.646 |
| 3MPG | 59.3 ± 5.3 | 56.3 ± 3.7 | 61.7 ± 4.2 | 0.660 |
| 3MPGS | 74.7 ± 6.8 | 71.4 ± 4.6 | 76.9 ± 5.2 | 0.754 |
| State U | 100.0 ± 8.2 | 96.8 ± 6.3 | 102.1 ± 8.2 | 0.871 |

*Data are presented as means ± standard error of the mean. There were no significant differences within groups.*

*ADP-stimulated (state 3) mitochondrial respiration was assessed upon octanoylcarnitine (O) or pyruvate (P), with malate (M) being present as supportive substrate. In both traces glutamate (G) and succinate (S) were added subsequently. Finally, maximal uncoupled respiration (state U) was assessed by titration of the chemical uncoupler FCCP.*

**Table S2.** Protein expression of OXPHOS complexes in lean, overweight / obese, and T2DM subjects

|  | Lean  (n=10) | Overweight or Obese  (n=15) | T2DM  (n=12) | P-value |
| --- | --- | --- | --- | --- |
| OXPHOS complex I | 0.954 ± 0.112 | 0.870 ± 0.116 | 1.200 ± 0.182 | 0.230 |
| OXPHOS complex II | 1.001 ± 0.107 | 0.848 ± 0.080 | 1.189 ± 0.183 | 0.160 |
| OXPHOS complex III | 1.056 ± 0.061 | 0.955 ± 0.069 | 1.010 ± 0.099 | 0.667 |
| OXPHOS complex IV | 0.979 ± 0.050 | 0.977 ± 0.057 | 1.046 ± 0.073 | 0.675 |
| OXPHOS complex V | 0.994 ± 0.067 | 0.978 ± 0.086 | 1.032 ± 0.098 | 0.901 |

*Data are presented as means ± standard error of the mean. There were no significant differences within groups.*

**Table S3.** Left ventricular function parameters of lean, overweight / obese, and T2DM subjects

|  | Lean  (n=10) | Overweight / Obese  (n=12) | T2DM  (n=10) | P-value |
| --- | --- | --- | --- | --- |
| End diastolic volume (ml) | 185.1 ± 24.4 | 158.1 ± 9.7 | 184.9 ± 27.4 | 0.561 |
| End diastolic volume / BSA (ml/m^2^) | 99.7 ± 11.7 | 77.3 ± 5.0 | 89.4 ± 12.3 | 0.272 |
| End systolic volume (ml) | 65.5 ± 10.5 | 58.2 ± 8.7 | 80.9 ± 23.9 | 0.562 |
| End systolic volume / BSA (ml/m^2^) | 35.1 ± 5.2 | 28.2 ± 4.1 | 38.9 ± 11.1 | 0.556 |
| Stroke volume (ml) | 119.7 ± 14.7 | 99.9 ± 7.1 | 103.9 ± 6.5 | 0.342 |
| Stroke volume / BSA (ml/m^2^) | 64.6 ± 7.1 | 49.1 ± 4.1 | 50.6 ± 2.5 | 0.064 |
| Ejection fraction (%) | 65.3 ± 2.2 | 64.4 ± 3.9 | 62.1 ± 5.0 | 0.842 |
| End diastolic mass (g) | 186.4 ± 15.7 | 176.4 ± 10.0 | 171.4 ± 14.4 | 0.732 |
| End diastolic mass / BSA (g/m^2^) | 101.0 ± 7.4 | 85.7 ± 4.0 | 83.0 ± 5.7 | 0.077 |
| End systolic mass (g) | 202.2 ± 15.9 | 173.2 ± 19.0 | 192.8 ± 14.9 | 0.469 |
| End systolic mass / BSA (g/m^2^) | 109.7 ± 7.5 | 82.7 ± 8.4 *^a^* | 93.2 ± 5.6 | 0.048 |
| Cardiac output (L/min) | 7.5 ± 0.9 | 5.4 ± 0.7* | 6.1 ± 0.9* | 0.196 |

*Data are presented as means ± standard error of the mean, or as proportions (%). BSA = body surface area. Ventricular mass is measured excluding papillary muscle mass. Significant differences between the groups were found for the indexed end systolic mass, and in particular between the overweight or obese and lean subjects.*

*^a^ Overweight or obese subjects significantly different from lean subjects. * Cardiac output was only measured in 8 out of the 12 overweight / obese subjects, and 7 out of the 10 T2DM subjects.*
