# Supplementary material for: PCr/ATP ratios and mitochondrial function in the heart. A comparative study in humans
Source: Sci Rep. 2023 May 23;13:8346. doi: 10.1038/s41598-023-35041-7 (PMC10205750; doi:10.1038/s41598-023-35041-7)
Supplement: Supplementary file 2 — Supplementary Figures. [file 41598_2023_35041_MOESM2_ESM.docx]

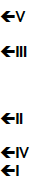

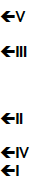

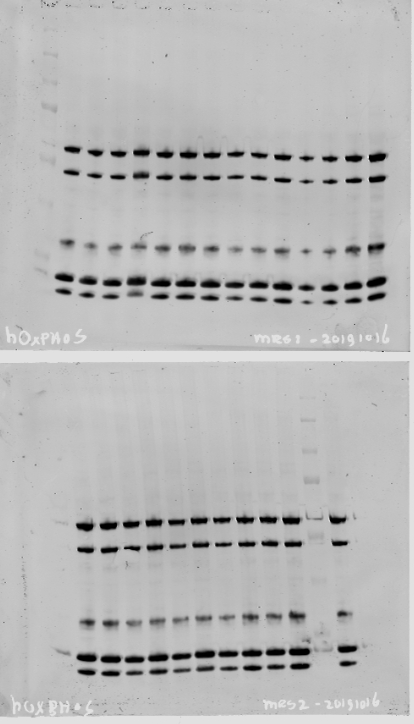


*Supplemental Figure 1. Uncropped Western Blots of the different complexes of the respiratory chain (Complex 1-5) with loading control and reference lines (lane 1 and 4 upper panel and lane 1 and 12 lower panel).*


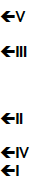

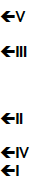

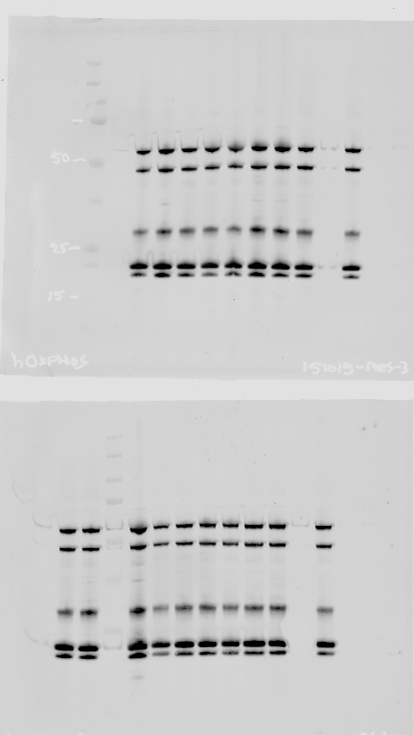


*Supplemental Figure 2. Uncropped Western Blots of the different complexes of the respiratory chain (Complex 1-5) in duplicates per subject with loading control and reference lines (lane 9 upper panel and lane 3 and 11 lower panel).*
